# Accurate emotion recognition using Bayesian model based EEG sources as dynamic graph convolutional neural network nodes

**DOI:** 10.1038/s41598-022-14217-7

**Published:** 2022-06-18

**Authors:** Shiva Asadzadeh, Tohid Yousefi Rezaii, Soosan Beheshti, Saeed Meshgini

**Affiliations:** 1 Department of Biomedical Engineering, Faculty of Electrical and Computer Engineering, University of Tabriz, Tabriz, Iran; 2 Department of Electrical and Computer Engineering, Ryerson University, Toronto, Canada

**Keywords:** Biomedical engineering, Emotion

## Abstract

Due to the effect of emotions on interactions, interpretations, and decisions, automatic detection and analysis of human emotions based on EEG signals has an important role in the treatment of psychiatric diseases. However, the low spatial resolution of EEG recorders poses a challenge. In order to overcome this problem, in this paper we model each emotion by mapping from scalp sensors to brain sources using Bernoulli–Laplace-based Bayesian model. The standard low-resolution electromagnetic tomography (sLORETA) method is used to initialize the source signals in this algorithm. Finally, a dynamic graph convolutional neural network (DGCNN) is used to classify emotional EEG in which the sources of the proposed localization model are considered as the underlying graph nodes. In the proposed method, the relationships between the EEG source signals are encoded in the DGCNN adjacency matrix. Experiments on our EEG dataset recorded at the Brain-Computer Interface Research Laboratory, University of Tabriz as well as publicly available SEED and DEAP datasets show that brain source modeling by the proposed algorithm significantly improves the accuracy of emotion recognition, such that it achieve a classification accuracy of 99.25% during the classification of the two classes of positive and negative emotions. These results represent an absolute 1–2% improvement in terms of classification accuracy over subject-dependent and subject-independent scenarios over the existing approaches.

## Introduction

In human daily life, emotions affect interactions, interpretations, and decision-making^1^. In addition, information about emotional states is essential for a more natural human–computer interface. In order to reduce the gap between human–machine interactions, the design of emotion recognition systems has been considered as a major research field in recent decades^2^. This area is considered as the intersection of artificial intelligence and human communication analysis. Face expressions and speech are mostly used to convey people's emotional states in daily life. However, these situations can be intentionally changed. Therefore, the use of this information will likely lead to the false classification of emotional states^3,4^. Electroencephalography (EEG) as a non-invasive physiological signal is suitable for direct measurement of the electrical activity of the brain in an emotional state. Hence, the study of these signals makes it possible to truly detect human emotions^5^. Despite the high temporal resolution of the EEG signal, the low spatial resolution of this signal poses a challenge when used in studies of functional brain activity. In order to increase the spatial resolution of EEG signal, this information is mapped from the sensor space to the space of brain sources. However, due to the limitations in the use of sensors, the number of brain sources is always more than the number of sensors^6^. This issue converts EEG signal mapping problem to an under-determined problem. EEG source imaging (ESI) becomes possible by solving an ill-posed inverse problem (Fig.‌‌ 1)^7^. ESI is a computational method for three-dimensional source localization of electrical activity in the cerebral cortex in the brain volume, also called EEG source localization. The estimation accuracy of this method depends on the choice of the head model and the inverse solution. The current due to postsynaptic potentials is propagated simultaneously by the pyramidal neurons according to Poisson equations, but this propagation is not homogeneous. High resistance of the skull weakens this current. This attenuation must be modeled in the calculations. MRI is used to determine the thickness of the skull and the resulting local conductivity properties. These properties are taken into account in the lead field to determine the relationship between electrical activity at a particular electrode and the activity of various sources in the brain. Accuracy in determining this lead field leads to increased source localization accuracy. Among the various ways to induce emotions, such as watching movies, watching images, listening to music is a better approach to stimulate brain, because sensory content of music directly reaches the audience and does not require translation or another medium^8,9^.

**Figure 1:**
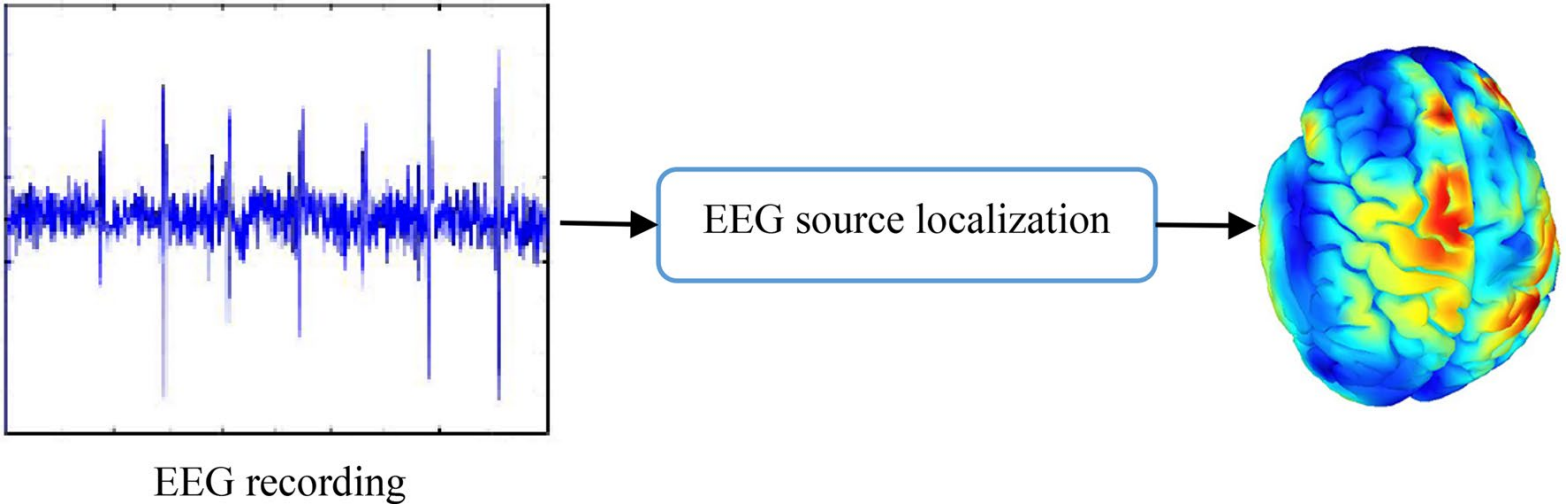
Source localization procedure of emotional EEG signal (using drawing canvas of Microsoft office word 2016 https://www.microsoft.com/en-us/download/details.aspx?id=51791 and Brainstorm version 3.211110 https://neuroimage.usc.edu/bst/download.php).

In^10^, the spatial and temporal distribution of the emotional EEG signal is calculated using the Independent Component Analysis (ICA) algorithms on the results of the standard low-resolution electromagnetic tomography (sLORETA) algorithm. In this study, specific independent components (ICs) were identified for listening to a piece of music and scales. Significant differences were observed between these ICs and the ICs calculated for rest time EEGs^10^. Active brain regions were calculated using low-resolution electromagnetic tomography (LORETA) in^11^. Hjorth parameters, power spectrum density, and wavelet are used as properties extracted from this data to classify emotions using the support vector machine classification (SVM) method. 57.30% accuracy has been obtained for this method. The accuracy score for this method in another research^12^ was reported 85.92%. The study of the effect of age on neural activation and response to various emotional stimuli in^13,14^ showed that aging affects the limbic area and thus changes emotional processing and N170 amplitude. In this study, the database of facial emotion images (POFA)^15^, which includes 110 black and white images of facial expressions, was used as emotional stimuli. The brain areas involved during emotional interference conditions have been investigated in^16^. The brain source activities were computed using sLORETA. Considerable decreased activity [p < 0.05/66] with respect to baseline are observed in Eighteen gyri in face-word interference and fifty-four gyri in word-face interference, respectively^16^. To detect EEG emotions, a dynamic convolutional neural network (DGCNN) is presented in^17^. The DREAMER dataset (a database for detecting emotions through EEG and ECG signals)^18^ and Shanghai Jiao Tong University (SJTU) emotion EEG dataset (SEED)^19^ have been used to evaluate this method. The results show a mean accuracy of 86.23%, 84.54%, and 85.02% for capacity, arousal, and dominance classification, respectively. The gender differences effect on EEG spectral power and source locations is evaluated in^20^.

Watching emotional music videos is applied as emotional stimuli in this research. In another study^21^, a combined technique of electrode frequency distribution maps (EFDMs) with short Fourier transform (STFT) was proposed. In order to classify emotions, a deep convolutional neural network based on residual block (CNN) has been utilized in this approach. The average classification score of this technique has been obtained 90.59% and 82.84% for SEED dataset and database of emotion analysis using physiological signals (DEAP)^22^, respectively. Researchers in^23^ used the Wigner-Will quasi-distribution (SPWVD) to convert filtered EEG signals into images. These images were intended as input to AlexNet, ResNet50 and VGG16, along with customizable CNN. The reported results show 90.98%, 91.91%, 92.71% and 93.01% accuracy for AlexNet, ResNet50, VGG16, and CNN, respectively. An instance-adaptive graph (IAG) approach has been suggested in^24^, in which sparse graphical representations of input EEG data have been constructed. According to the results, the accuracy of this method is 86.30%. A regularized graph neural network (RGNN) has been offered in^25^ for the emotional EEG classification. Accuracy of 73.84% on the SEED-IV dataset is achieved and 85.30% on EEG SEED dataset for this procedure. In^26^, channel-wise features are applied as the input of two-layer stacked short-term memory (LSTM). Accuracies of 98.93% and 99.10% during the two-class classification of valence and arousal in the DEAP dataset and an accuracy of 99.63% during the three-class classification on the SEED dataset have been attained, respectively.

Examining existing methods for emotion recognition reveals that the high spatial resolution of the EEG signal is crucial for extracting sufficient information in the feature selection and extraction process for emotion recognition. As mentioned above, the mapping of the EEG signal from the scalp sensor space to the brain source space can accurately show the pattern of the brain areas involved during emotional stimuli. The relationship between different brain areas can be determined based on these source signals relation. The pattern of brain activity during emotional stimulation is determined by creating a graph based on the relationship of the brain source. These graphs are used to separate different emotions. In study^17^, raw EEG signal information is used as input to the DGCNN network, but in our proposed method, the raw EEG signal is first given to an EEG source localization algorithm—Bayesian model based on the Bernoulli-Laplace prior method—as input. The output of this algorithm contains spatio-temporal information of emotional EEG sources. Accordingly, in addition to temporal information, topographic and spatial information of electrical activity of the brain is entered in the recognition process. This information is encoded in a graph. Relationships between brain sources extracted from the localization algorithm used to weight the adjacency matrix of this graph. The results are used in the DGCNN algorithm to classify emotions. The potentials recorded in the electrodes actually represent the superposition of these brain source activities. As a result, it is clear that the information obtained from the localization algorithms is more accurate and efficient than the raw EEG signal information.

In this study, features obtained from extracted Bernoulli-Laplace-based Bayesian model sources are considered as the signal of dynamical graph convolutional neural networks (DGCNN) nodes. By encoding the inter-source relations of EEG source signals in the adjacency matrix, the pattern of activity in different brain areas is used to increase the accuracy of emotion classification. This algorithm allows the classification of unseen emotional EEG signals into negative and positive emotional classes.

The main sections of this study are summarized as follows: In Section “Mathematical background”, Mathematical background of EEG Source localization and dynamical graph convolutional neural networks (DGCNN) have been introduced. Then, the proposed approach for emotional states classification has been provided in Section “Emotional EEG source recognition based on DGCNN”. In the “Simulation results” section, the results of the proposed method are explained. Finally, the results will be discussed.

## Mathematical background

In this section, the basic theory of EEG source localization and dynamical graph convolutional neural networks will be presented.

### EEG source localization

EEG source localization method provides spatio-temporal information about the activity of different areas of the brain. Brain source localization improves the non-invasive detection of functional, mental, and even physiological abnormalities related to the brain in clinical applications^27^. In these methods, the sources are considered as several discrete magnetic dipoles in the three-dimensional space of the brain. One of the most common methods in this field is the LORETA method. The basic hypothesis in this method is that the current density of brain source at any point in the cortex is close to the average current density of its neighbors. A major problem in this method is the low spatial resolution and the blurring and scattering noise of the point sources of the images^28^. In order to solve this problem, using the current density standardization hypothesis, the sLORETA method has been proposed as a generalization of the LORETA method^29^. Since the electric potential at any point on the scalp is a linear combination of the dipole amplitudes of the brain sources, therefore, the relationship between the potential in the scalp and the dipole amplitudes of the sources is defined as follows^30,31^:

1 $$ {\mathbf{y}} \, = \, {\mathbf{Hx}} \, + {\mathbf{ e}} $$

where, ${\mathbf{y}} \in {\mathbb{R}}^{N}$ is the EEG data of *N* electrodes and the amplitudes of *M* dipoles in the 3D spatial space is shown by ${\mathbf{x}} \in {\mathbb{R}}^{M}$. The *N* × *M* lead field matrix **H** models the propagation of the electromagnetic field from the sources to the sensors and the noise of recorded EEG data is considered as an additive white Gaussian noise **e**^32,33^.

As mentioned above, the inverse problem is an under-determined problem due to the limited placement of electroencephalogram sensors and a large number of brain sources. This imposes more constraints on achieving a unique solution. Proper regulation is usually required to solve an ill-posed inverse problem. Solutions that are considered the usual $l_{2}$ norm have low computational complexity. However, in several cases, it is believed that the actual activity of the brain is concentrated in several focal areas. In such situations, the $l_{2}$ norm creates overestimating problem of active space areas. To solve this problem, the promotion of sparse solutions is proposed, for example, based on $l_{1}$ norm that can be easily controlled by optimization techniques. In^34^, it is considered to use a $l_{0} + l_{1}$ norm to apply sparse source activity (ensuring that a small number of non-zero elements are present in the solution) while regulating the non-zero amplitudes of the solution. More precisely, the norm limits the amplitude values of non-zero elements while the pseudonorm controls their position. Using Bernoulli–Laplace prior, the hybrid $l_{0} + l_{1}$ norm is introduced in the Bayesian framework. The proposed Bayesian model uses the Markov chain Monte Carlo sampling technique to estimate the model hyperparameters. It has been proven that this model is in favor of sparsity. It is very common to consider an additive white Gaussian noise with variance $\sigma_{n}^{2}$ in EEG analysis^30^.

${{\varvec{\uptheta}}} \, = \, \left\{ {{\mathbf{x}},\sigma_{n}^{2} } \right\}$ is unknown parameter vectors related to the proposed model (1). Priors of these parameters for Bayesian inference are given as follow:

1. Dipole Amplitudes Prior: A $l_{0} + l_{1}$ regularization using Bernoulli-Laplace prior distribution for each ${\mathbf{x}}$ vector element is introduced similar to Bayesian to encourage sparse solutions whose non-zero elements have small amplitudes. The corresponding pdf for the *i*th element of ${\mathbf{x}}$ is
  2 $$ f(x_{i} |\omega ,\lambda ) \, = \, (1 - \omega )\delta (x_{i} ) \, + \frac{\omega }{2\lambda }exp\left( { - \frac{{|x_{i} |}}{\lambda }} \right) $$
  where the parameter of the Laplace distribution is $\lambda$, the Dirac delta function is $\delta \left( . \right)$. ω as a weight balances the effects of the Laplace distribution and the Dirac delta function.
2. The Noise Variance Prior: A noninformative Jeffrey’s prior is considered for the noise variance:
  3 $$ f(\sigma_{n}^{2} ) \propto \frac{1}{{\sigma_{n}^{2} }}1_{{{\mathbb{R}}^{ + } }} (\sigma_{n}^{2} ) $$
  where $1_{{{\mathbb{R}}^{ + } }} (\xi ) \, = \, 1{\text{ if }}\xi \in {\mathbb{R}}^{ + }$ and 0 otherwise. This is a very common choice for a noninformative prior^35^. Attend that a more informative prior distribution of signal-to-noise ratio can also be considered.

The hyperparameter vector of the previous priors is ${{\varvec{\Phi}}} \, = \, \left\{ {\omega ,\lambda } \right\}$.

The joint posterior distribution of the model can be represented by considering the previously introduced likelihood and priors using the following hierarchical construction:

4 $$ f\left( {{{\varvec{\uptheta}}},{{\varvec{\Phi}}}|{\mathbf{y}}} \right) \propto f\left( {{\mathbf{y}}|{{\varvec{\uptheta}}}} \right)f\left( {{{\varvec{\uptheta}}}|{{\varvec{\Phi}}}} \right)f\left( {{\varvec{\Phi}}} \right) $$

where the model parameters and hyperparameters vector is $\left\{ {{{\varvec{\uptheta}}},{{\varvec{\Phi}}}} \right\}$. The Bayesian estimators of $\left\{ {{{\varvec{\uptheta}}},{{\varvec{\Phi}}}} \right\}$ cannot be calculated with simple closed-form declarations, because this posterior distribution has complexities. In order to sample the joint posterior distribution, a Markov chain monte carlo (MCMC) method can be used (4). This method uses the generated samples to build Bayesian estimators of the unknown model parameters. For this purpose, a Gibbs sampler^35^ is considered, which generates samples repeatedly from conditional distributions (4), i.e., from $f\left( {\sigma_{n}^{2} |{\mathbf{y}}, \, {\mathbf{x}}} \right), \, f\left( {\lambda |{\mathbf{x}}} \right), \, f\left( {\omega |{\mathbf{x}}} \right){\text{ and }}f\left( {x_{i} |{\mathbf{y}},{\text{ x}}_{ - i} ,\omega ,\lambda ,\sigma_{n}^{2} } \right).$

The likelihood and the prior distribution of **x** are used to calculate the conditional distribution of each signal element x_*i*_. This distribution can be defined as follows:

5 $$ f\left( {x_{i} |{\mathbf{y}},x{}_{ - i},\omega ,\lambda ,\sigma_{n}^{2} \, } \right) \, = \, \omega_{1,i} \delta \left( {x_{i} } \right) \, + \, \omega_{2,i} {\mathcal{N}}_{ + } \left( {\mu_{i, + } ,\sigma_{i}^{2} } \right) + \, \omega_{3,i} {\mathcal{N}}_{ - } \left( {\mu_{i, - } ,\sigma_{i}^{2} } \right) $$

where the truncated Gaussian distributions on ${\mathbb{R}}^{ + }$ and ${\mathbb{R}}^{ - }$ are shown using ${\mathcal{N}}_{ + }$ and ${\mathcal{N}}_{ - }$, respectively. The vector **x** can be decomposed on the orthonormal basis B = {**n**_1_, … ,**n**_M_} such that ${\mathbf{x}} \, = \, {\tilde{\mathbf{x}}}_{ - i} \, + \, x_{i} {\mathbf{n}}_{i}$ where ${\tilde{\mathbf{x}}}_{ - i}$ is obtained by setting the *i*th element of **x** to 0. Defining ${{\varvec{\upnu}}}_{i} \, = \, {\mathbf{y}} \, - \, {\mathbf{H\tilde{x}}}_{ - i}$ and ${\mathbf{h}}_{i} \, = \, {\mathbf{Hn}}_{i}$, the weights are defined as

6 $$ \omega_{l,i} \, = \frac{{u_{l,i} }}{{\sum\limits_{l = 1}^{3} { \, u_{l,i} } }} $$

where

7 $$ u_{1,i} \, = \, 1 \, - \, \omega , \, u_{2,i} \, = \frac{\omega }{2\lambda }exp \, \left( {\frac{{\left( {\mu_{i}^{ + } } \right)^{2} }}{{2\sigma_{i}^{2} }}} \right) \sqrt {2\pi \sigma_{i}^{2} } C\left( {\mu_{i}^{ + } ,\sigma_{i}^{2} } \right), \, u_{3,i} \, = \frac{\omega }{2\lambda }exp \, \left( {\frac{{\left( {\mu_{i}^{ - } } \right)^{2} }}{{2\sigma_{i}^{2} }}} \right) \sqrt {2\pi \sigma_{i}^{2} } C\left( { - \mu_{i}^{ - } ,\sigma_{i}^{2} } \right) \, $$

and

8 $$ \sigma_{i}^{2} = \frac{{\sigma_{n}^{2} }}{{\left\| {{\mathbf{h}}_{i} } \right\|^{2} }}, \, \mu_{i}^{ + } = \sigma_{2}^{i} \left( {\frac{{{\mathbf{h}}_{i}^{T} {\mathbf{v}}_{i} }}{{\sigma_{n}^{2} }} - \frac{1}{\lambda }} \right), \, \mu_{i}^{ - } = \sigma_{2}^{i} \left( {\frac{{{\mathbf{h}}_{i}^{T} {\mathbf{v}}_{i} }}{{\sigma_{n}^{2} }} + \frac{1}{\lambda }} \right), \, C(\mu ,\sigma^{2} ) \, = \frac{1}{2}\left[ {1 \, + erf\left( {\frac{\mu }{{\sqrt {2\sigma^{2} } }}} \right)} \right] \, $$

### Dynamical graph convolutional neural network

Network data can be easily modeled as a graph signal. In this situation, the fundamental network topology is demonstrated using a graph. Data values are consecrated to the graph nodes. An undirected graph ${\mathcal{G}} = ({\mathcal{V},\mathcal{D}},{\mathbf{W}})$ with node set ${\mathcal{V}} = \left\{ {1,...,M} \right\}$, edge set ${\mathcal{D}} \subseteq {\mathcal{V}} \times {\mathcal{V}}$ and ${\mathbf{W}} \in {\mathbb{R}}^{M \times M}$ define an weighted adjacency matrix that explains the connections between any two nodes in ${\mathcal{V}}$. $w_{ij}$ is the entry of ${\mathbf{W}}$ in the *i*-th row and *j*-th column. The set of nodes that share an edge with node *i* is called the neighborhood of node $i \, \in \, {\mathcal{V}}$, which are defined as $C_{i} = \left\{ {j \in {\mathcal{V}}:(j,i) \in {\mathcal{D}}} \right\}$.

A common signal processing method for graph data operation is graph convolution or spectral graph filtering, in which graph Fourier transform (GFT)^36^ is typically used. The Laplacian matrix of the graph ${\mathcal{G}}$ is defined using **L**. **L** can be represented as follow:

9 $$ {\mathbf{L}} = {\mathbf{S}} - {\mathbf{W}} \in {\mathbb{R}}^{M \times M} , $$

where *i*th diagonal element of ${\mathbf{S}} \in {\mathbb{R}}^{M \times M}$ diagonal matrix can be computed by ${\mathbf{S}}_{ii} = \sum\nolimits_{j} {w_{ij} }$. The GFT of a given signal ${\mathbf{x}} \in {\mathbb{R}}^{M}$ is represented as:

10 $$ {\hat{\mathbf{x}}} = {\mathbf{U}}^{T} {\mathbf{x}}, $$

where the transformed signal in the frequency domain is defined by ${\hat{\mathbf{x}}}$. The singular value decomposition (SVD) of the graph Laplacian matrix **L** is an orthonormal matrix **U** as follow^37^:

11 $$ {\mathbf{L}} = {\mathbf{U\Lambda U}}^{T} , $$

By considering (10), the inverse GFT can be declared as follows:

12 $$ {\mathbf{x}} = {\mathbf{UU}}^{T} {\mathbf{x}} = {{\mathbf{U}\hat{\mathbf x}}}. $$

For the two signals **x** and ${\mathbf{z}}$, the convolution on the graph $*_{{\mathcal{G}}}$ can be defined as follows^38^:

13 $$ {\mathbf{x}} *_{{\mathcal{G}}} {\mathbf{z}} = {\mathbf{U}}(({\mathbf{U}}^{T} {\mathbf{x}}) \odot ({\mathbf{U}}^{T} {\mathbf{z}})), $$

where $\odot$ shows Hadamard's product in terms of element.

The optimal adjacency matrix ${\mathbf{W}}^{ * }$ can be learned. The spatial filtering ${\text{g}} ({\mathbf{L}}^{ * } )$ defines the graph convolution of **x** signal with the vector ${\mathbf{U}}^{ * } {\mathbf{g}}({{\varvec{\Lambda}}}^{ * } )$, which can be demonstrated as follows:

14 $$ {\mathbf{z}} = {\text{g}} ({\mathbf{L}}^{ * } ){\mathbf{x}} = {\mathbf{U}}^{ * } {\mathbf{g}}({{\varvec{\Lambda}}}^{ * } ){\mathbf{U}}^{ * T} {\mathbf{x}}, $$

where ${\text{g}} ({{\varvec{\Lambda}}})$ is demonstrated as

15 $$ {\mathbf{g}}({{\varvec{\Lambda}}}) \, = \left[ {\begin{array}{*{20}c} {{\text{g}} (\lambda_{0} )} & \ldots & 0 \\ \vdots & \ddots & \vdots \\ 0 & \cdots & {{\text{g}} (\lambda_{N - 1} )} \\ \end{array} } \right]. $$

where the ${\mathbf{L}}^{ * }$ can be computed based on (9) using ${\mathbf{W}}^{ * }$, and ${{\varvec{\Lambda}}}^{ * } = {\text{diag}} ([\lambda_{0}^{ * } {,} \cdots , \, \lambda_{N - 1}^{ * } ])$ is a diagonal matrix, whereas direct calculation of ${\mathbf{g}}({{\varvec{\Lambda}}}^{ * } )$ expression is difficult, we use, e.g. the *Kψ* order Chebyshev polynomials to fastly calculate the polynomial expansion of ${\mathbf{g}}({{\varvec{\Lambda}}}^{ * } )$ as follow^38^:

16 $$ {\mathbf{g}}({{\varvec{\Lambda}}}^{ * } ) \, = \sum\limits_{k = 0}^{K - 1} {\theta_{k} T_{k} ({\tilde{\mathbf{\Lambda }}}^{ * } )} , $$

where the following recursive expressions can be used to *T*_*k*_ (*x*) recursively calculation. $\theta_{k}$ is the coefficient of Chebyshev polynomials.

17 $$ \left\{ {\begin{array}{*{20}c} {T_{0} (x) = \, 1, \, T_{1} \left( x \right){ = }x,} & {} \\ {T_{k} (x) \, = 2xT_{k - 1} (x) - T_{k - 2} (x),} & {k \ge 2.} \\ \end{array} } \right. \, $$

Therefore, (16) is used to rewrite the convolution graph operation of (14) as follow:

18 $$ {\mathbf{z}} = \sum\limits_{k = 0}^{K - 1} {\theta_{k} T_{k} ({\tilde{\mathbf{L}}}^{ * } ){\mathbf{x}}} , $$

where $${\tilde{\mathbf{L}}}^{ * } = \, {{2{\mathbf{L}}^{*} } \mathord{\left/ {\vphantom {{2{\mathbf{L}}^{*} } {\lambda_{max}^{ * } }}} \right. \kern-\nulldelimiterspace} {\lambda_{max}^{ * } }} - {\mathbf{I}}_{M} .$$

The backpropagation (BP) method is used to iteratively optimize the optimal network parameters, in which the network parameters update until the optimal or suboptimal solutions are attained. Thus, a loss function is expressed based on cross-entropy cost. In order to dynamically learn the optimal adjacency matrix ${\mathbf{W}}^{ * }$ of the DGCNN model in the BP method, we must calculate the partial derivative of the loss function relative to ${\mathbf{W}}^{ * }$. After that, the updating formula of the optimal adjacency matrix ${\mathbf{W}}^{ * }$ can be expressed as:

19 $$ {\mathbf{W}}^{ * } = \, (1 - \rho ){\mathbf{W}}^{ * } + \rho \, \frac{\partial Loss}{{\partial {\mathbf{W}}^{ * } }}, $$

where the learning rate of the network is shown by *ψψ*.

## Emotional EEG source recognition based on DGCNN

In this section, how to extract signals from the brain sources of emotions, the use of the DGCNN algorithm to classify the types of emotional states, and detailed information about the data used in this study are described in detail.

### Proposed classification algorithm using DGCNN and Bayesian model based emotional EEG source

**Figure 2:**
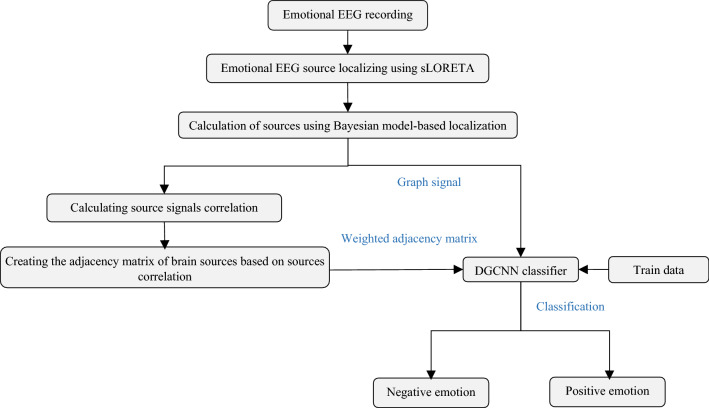
Flowchart of the proposed method (using drawing canvas of Microsoft office word 2016 https://www.microsoft.com/en-us/download/details.aspx?id=51791).

Considering the challenges of feature selection and extraction in previous methods and the need to increase the accuracy of classifying both positive and negative emotions, this section presents a method based on EEG source localization and graph theory. To this end, Fig. 2 shows a block-diagram of the proposed method for classifying two emotional classes:

- *Emotional EEG source localization using Bernoulli-Laplace-based Bayesian model*: The brain sources that generate the EEG signal are calculated using Bernoulli-Laplace-based Bayesian model algorithm. This algorithm is initialized using the sLORETA method.
- *Graph generation*: In the proposed method, a graph signal on the top of each graph node is obtained based on each extracted source signal. The graph adjacency matrix is also weighted based on the calculated correlation between the extracted source signals.
- *Graph pattern classification using the DGCNN algorithm:* The weighted graph adjacency matrix, the graph corresponding to the extracted source signals, is given as input to the DGCNN algorithm for recognizing and classifying emotions.

**Figure 3:**
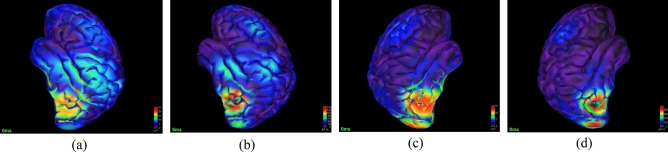
The differences of active brain sources for subject 1 during positive and negative emotional stimulation in cortical level and BA. lateral view of active brain areas for subject 1 during positive emotional stimulation (**a**) using the sLORETA (**b**) using the Bayesian model based on Bernoulli-Laplace prior. lateral view of brain active areas for subject 1 during negative emotional stimulation (**c**) using the sLORETA (**d**) using the Bayesian model based on Bernoulli–Laplace prior (Brainstorm version 3.211110 https://neuroimage.usc.edu/bst/download.php).

In this study, active areas of the brain during two kinds of emotional stimuli are identified using the proposed Bayesian model based on Bernoulli-Laplace prior. The sLORETA method is applied to initialize the source signals in this algorithm. To calculate the results of the sLORETA algorithm, we use the Colin27 brain atlas model from the Montreal Neurological Institute (MNI) and the OpenMEEG BEM head model^39,40^. The localization solution space is surrounded by the gray matter of the cortex. A resolution of 5 mm (mm/voxel) with 5614 voxels at MNI coordinates is used for this space in localization. If the number of vertices in the space of the localization solution increases, the recognition accuracy of the active areas during emotion induction increases. The differences between the active brain sources for recorded dataset in the Bradmann (BA) of cerebral cortex^41^ for sLORETA and Bayesian model based on Bernoulli-Laplace prior methods are shown in Fig. 3. The lateral view of the active brain areas for subject 1 during positive and negative emotional stimulation using the sLORETA method is shown in Fig. 3a and c, respectively. In addition, the lateral view of the active brain areas for subject 1 during positive and negative emotional stimulation using the Bayesian model based on Bernoulli–Laplace prior is presented in Fig. 3b and d, respectively.

**Figure 4:**
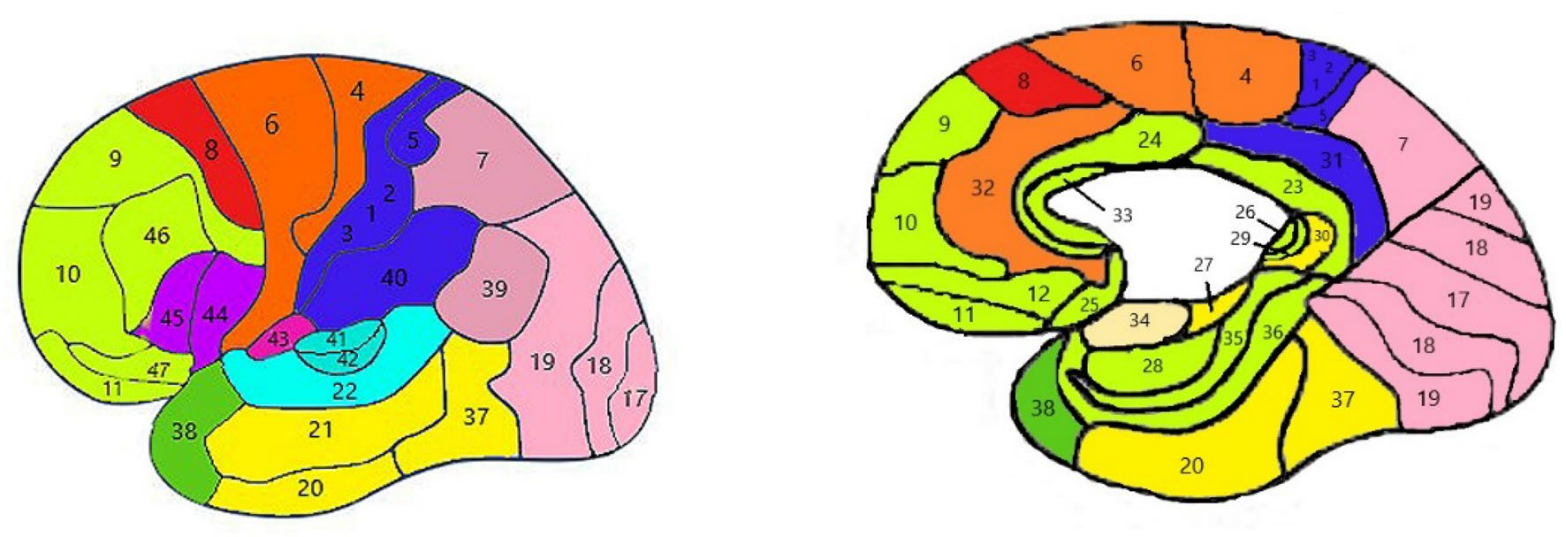
The labels of Brodmann areas (using Paint 3D version 6.1907.29027.0 https://apps.microsoft.com/store/detail/paint-3d/9NBLGGH5FV99?hl=en-us&gl=US).

In the results of sLORETA topographic images, the areas including the auditory cortex, lingual gyrus, and amygdala located in the lower and middle temporal cortex and the middle occipital cortex show the most activity during emotional stimulation of the brain. Considering the results of the sLORETA method, 26 Broadman regions are considered as the region of interest (ROI) for feature extraction. BA5, 6, 7, 9, 10, 11, 18, 19, 21, 22, 29, 37, 38, 39, and 40 with bilateral hemispheres are selected as ROI areas (Fig. 4).

**Figure 5:**
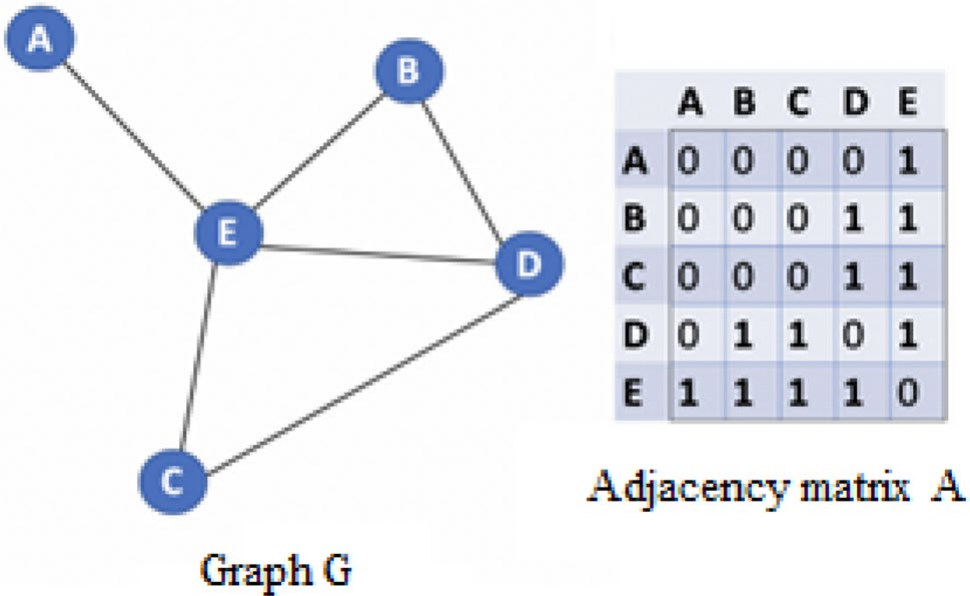
Example of a graph and its adjacency matrix^17^.

However, the Bayesian model based on Bernoulli–Laplace prior method concentrates the active areas and thus reduces the number of these areas. Unlike previous methods, this method simplifies the complex pattern of most active brain areas. Differences in the brain areas that are activated during positive and negative stimuli indicate that a spatial-information-aware classifier can be used to accurately classify emotions. In the proposed method, most activities are seen in BA 19, 37, 18 areas due to the induction of negative emotions and BA 20, 21, 22 due to the induction of positive emotions. Bayesian model based on Bernoulli–Laplace prior method calculates the current source density (CSD) for each voxel (in amperes in each region). In order to reduce the computational volume of the proposed method and identify a set of the powerful dipoles and their corresponding neighbors, we calculate the energy of all source signals and then choose all signal sources whose power is greater than 50% of the maximum power of the activity amplitude. The signals from each source are used as input to the classification algorithm. Sources with less than 50% of the maximum power are discarded to reduce computational complexity. From what has been said, it is clear that the formation of source signals graph can provide a pattern of different areas activity during an emotional stimulus to classify emotions. In this case, there will be a graph of brain sources for each emotional stimulus. For this purpose, an adjacency matrix that describes the relationships between nodes will be needed. In this matrix, if there is an edge from node *i* to node *j*, ${\mathbf{A}}_{ij} ,{\mathbf{A}}_{ji} = 1$, otherwise ${\mathbf{A}}_{ij} ,{\mathbf{A}}_{ji} = 0$ (Fig. 5).

The location of the vertices in the MNI coordinates is considered as graph nodes and the corresponding source signal is considered as the graph signal at the top of that node. In the proposed approach, the correlation between the two corresponding nodes signals of one edge is considered as the initial value of the graph edge weight. More precisely, the correlation between the $i$-th source ${\mathbf{x}}_{i}$ and the $j$-th source ${\mathbf{x}}_{j}$ can be computed as follows:

20 $$ w_{ij} = \frac{{\sum\limits_{t = 1}^{T} {\left( {{\mathbf{x}}_{it} - {\overline{\mathbf{x}}}_{i} } \right)\left( {{\mathbf{x}}_{jt} - {\overline{\mathbf{x}}}_{j} } \right)} }}{{\sqrt {\sum\limits_{t = 1}^{T} {\left( {{\mathbf{x}}_{it} - {\overline{\mathbf{x}}}_{i} } \right)^{2} } } \sqrt {\sum\limits_{t = 1}^{T} {\left( {{\mathbf{x}}_{jt} - {\overline{\mathbf{x}}}_{j} } \right)^{2} } } }}, \, $$

where $$ \, i,j \in \left\{ {1,...,M} \right\}{,}t \in \left\{ {1,...,T} \right\}$$.

**Figure 6:**
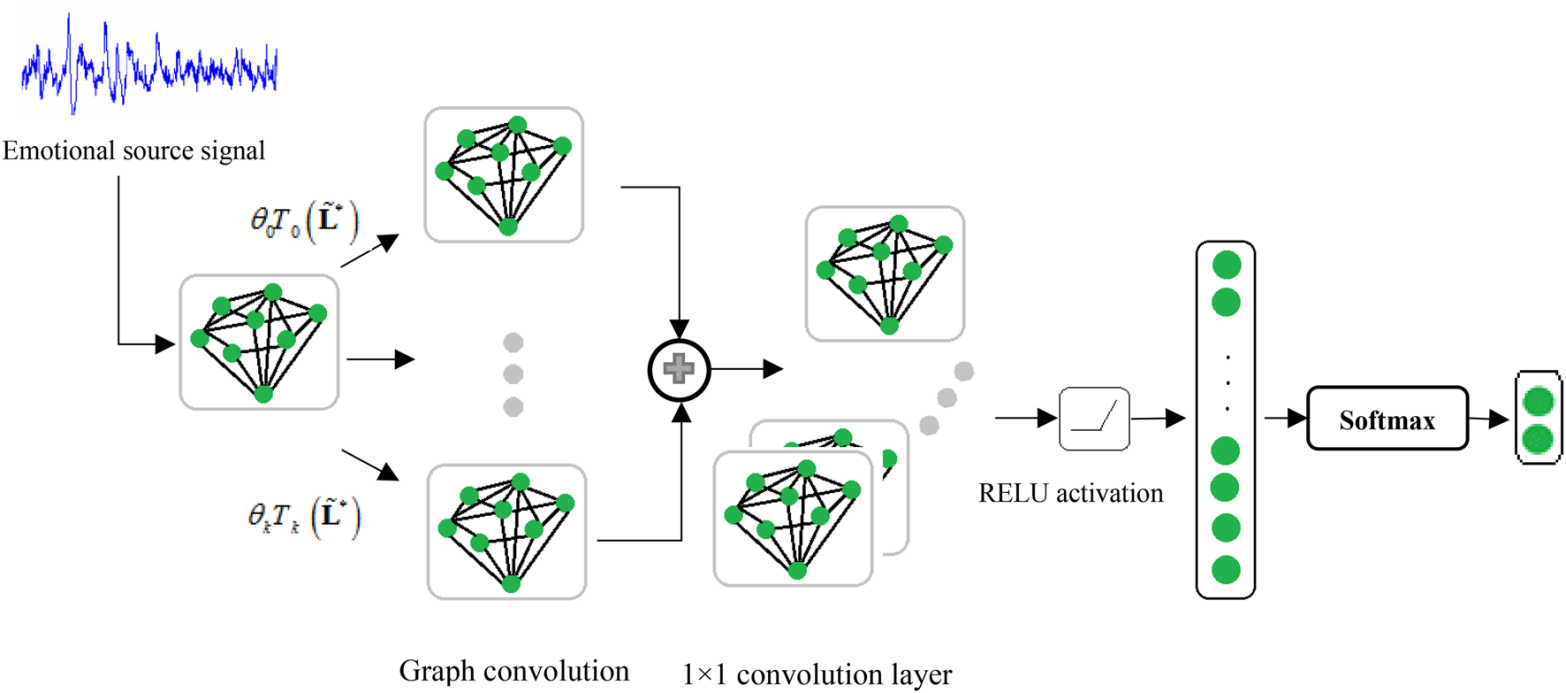
The framework of the DGCNN model for EEG emotion recognition, which consists of the graph convolutional operation, activation function and a fully connected layer. The inputs of the model are the EEG source signals, where each EEG source signal is represented as a node of the graph. The outputs are the predicted labels through softmax function.

Here, we define a threshold $\beta$, such that when $w_{ij} > \beta$, the $i$-th source is linked with the $j$-th source in the constructed graph $\mathcal{G}$. In this paper, a model based on graph-structured data is proposed to learn and classify the patterns of EEG source. In DGCNN, the adjacency matrix is updated with graph model parameters changes during model training to learn the relationships between EEG source signals according to (19), unlike the traditional graph convolutional neural network (GCNN) method, in which the adjacency matrix was determined before model training. This approach improves the classification results. In the proposed algorithm, the network parameters are frequently updated to achieve optimal or semi-optimal solutions according to (16). The structure of the proposed algorithm is indicated in Fig. 6, which includes the graph filtering layer, convolutional layers, and one fully connected layer. The detailed procedures of the proposed algorithm are summarized in Algorithm 1.
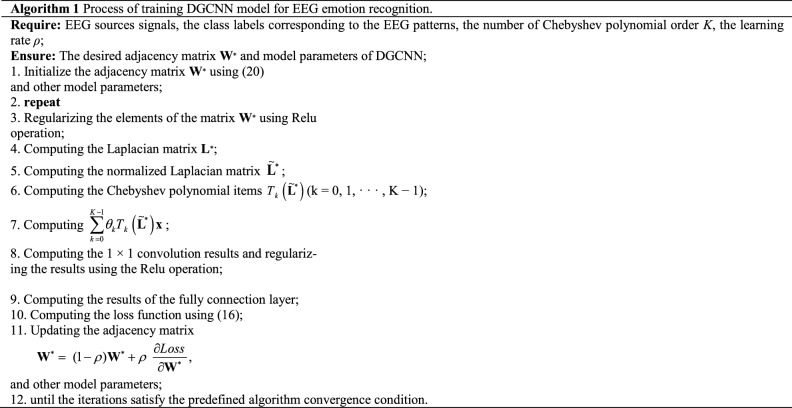

### Emotional EEG datasets

#### SEED dataset

In this data set, the EEG signal of 15 Chinese people (8 females and 7 males, age range: 23.27 ± 2.37) was recorded while watching 15 video clips. Chinese film clips with three types of emotions, i.e., negative, positive and neutralis shown for subjects.The sampling rate is 200 Hz. After watching each clip, participants immediately chose emotional labels that included positive, neutral, and negative attributes. A bandpass frequency filter from 0 to 75 Hz was applied. A hamming window with a specific duration with non-overlap was used to divide each signal into 8 data segments.

#### DEAP dataset

DEAP is a database containing physiological signals for analyzing emotions. EEG and environmental physiological signals were recorded by 32 healthy participants (16 males and 16 females, aged 19 to 37 years) while each watching 40 one-minute pieces of music videos. 32 active AgCl electrodes (placed according to International System 10–20) with a sampling rate of 512 Hz were used for EEG recording. This database includes peripheral nervous system signals: GSR, respiratory rate, skin temperature, electrocardiogram, blood volume by plethysmography, zygomatic and trapezoidal muscle electromyogram, and electrooculogram (EOG). The 32-channel EEG data sampling were reduced to 128 Hz and the EOG was removed by filtering 4.0–45.0 Hz from the data. And then, a 5 s hamming window with non-overlap was used to divide each signal into 12 data segments.

#### Recorded EEG

**Figure 7:**
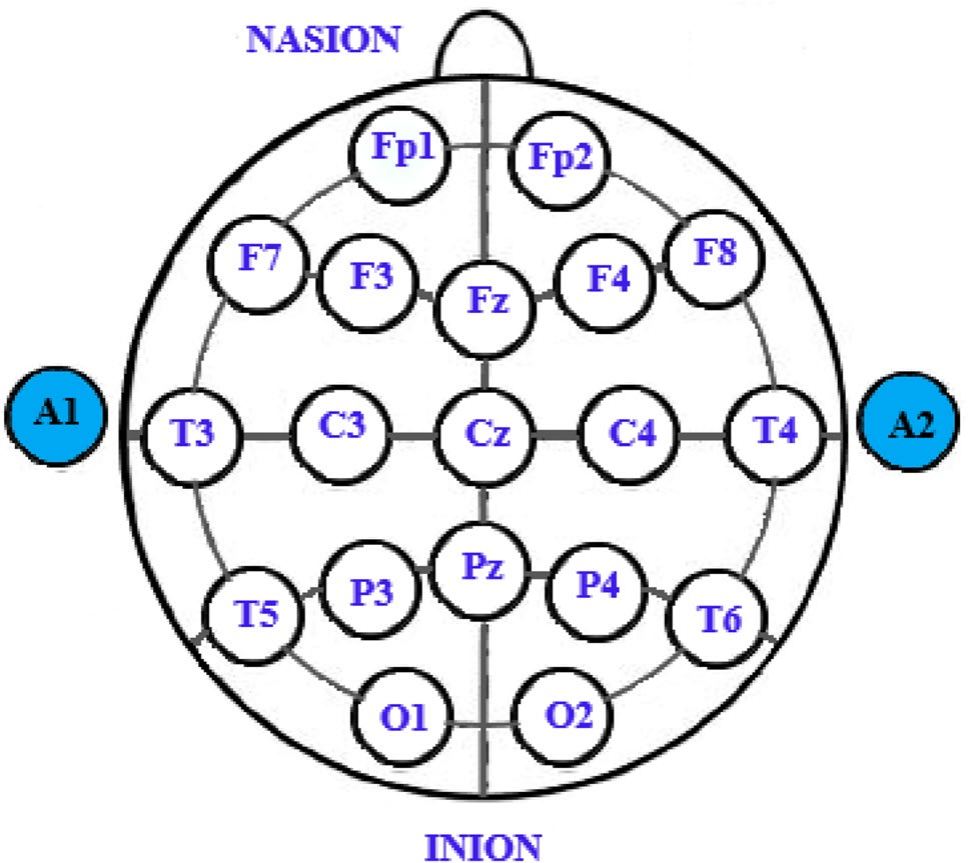
The schematic image of 21 electrodes on the head (using Paint 3D version 6.1907.29027.0 https://apps.microsoft.com/store/detail/paint-3d/9NBLGGH5FV99?hl=en-us&gl=US).

**Figure 8:**
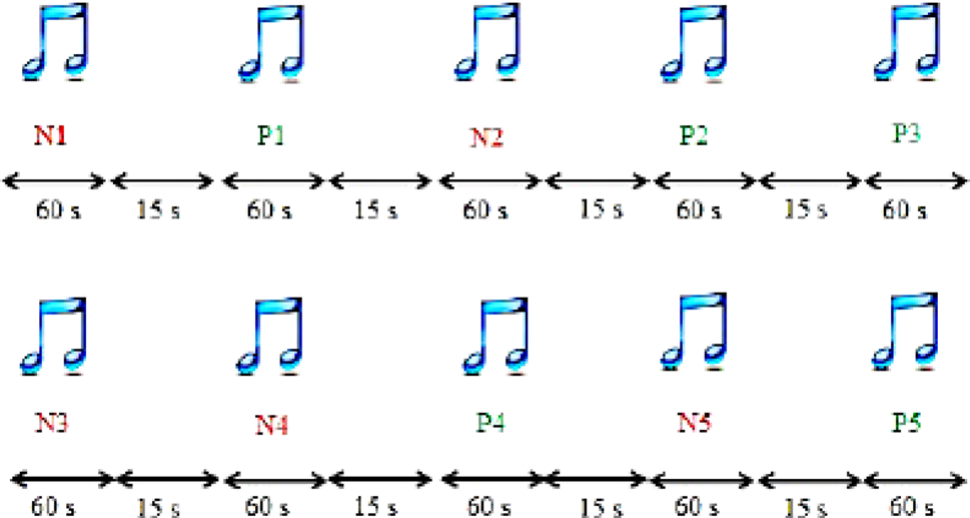
The procedure of musical stimulation to recognize emotions^43^, (Paper^43^ was published in IEEE Access under a Creative Commons 4.0 license).

In the database of the Brain-Computer Interface Research Laboratory, University of Tabriz, Iran, the EEG signals of 16 people without a history of mental illness (6 women and 10 men between 28 and 21 years old) were recorded while listening to emotional music^42,43^. The 21-channel Encephalan Medicom device was used to record the EEG signal. The sampling rate in this experiment is about 250 Hz. The international standard system 10–20 is utilized to arrange the electrodes on the head (Fig. 7). In the questionnaire version, the Self-Assessment Manikin (SAM)^44^ and in the test process, a 9-point test was used to assess positive and negative emotions. In addition, the participants^45^ completed the Beck Depression Inventory (BDI) questionnaire. The SAM results and description of the BDI test are presented in Table 1. Details of the selected music for each theme are demonstrated in Table 2. The sequence of how to play musical stimuli for participants is shown in Fig. 8. A fifteen-second silence is applied between the two pieces of music. An intermediate filter with cut-off frequencies of 0.5 and 70 Hz is used to extract useful EEG signal information. According to Fig. 8, the number of data related to the neutral class is less than the data of the positive and negative classes, which causes an imbalance between the data and may cause the problem of over-fitting. In addition, an imbalance between the data of each class leads to bias in the classification results and a decrease in accuracy. To solve this problem, using overlapping methods, all the corresponding epochs of each emotion are connected to form a long signal. Rectangular windows are then executed with a specific duration and overlap so that the number of epochs collected is equal to each of the emotion classes. In the proposed method for each channel, 5 min of recorded signal (as shown in Fig. 3) is selected for each emotion. In this case we have 2 data classes (negative and positive) with 75,000 sample points for each channel. The data is then split into 8-s intervals per channel, using the overlap technique to prevent over-fitting.

**Table 1:** Validation of individuals participating in the EEG signal recording process in order to identify positive and negative emotions^43^.

Subject number	Sex	Age	BDI	Mean valence for positive emotion	Mean arousal for positive emotion	Mean valence for negative emotion	Mean arousal for negative emotion	Validation result	Reason for subject removal
1	Male	25	16	9	9	1.8	1	Valid	–
2	Male	24	22	6.8	6.2	3.6	2	Invalid	Beck depression (22 > 21)
3	Female	27	19	6.2	7.4	4.2	4.6	Invalid	Mismatch of the control question in the SAM test
4	Male	24	4	7.4	7.6	2.4	2.6	Valid	–
5	Male	24	0	5.8	5	4.4	5.6	Invalid	Mismatch of the control question in the SAM test
6	Male	28	10	5.6	5.4	2	1.6	Invalid	Lack of desired induction in positive emotional class
7	Male	28	13	7.2	7.4	3.8	3.8	Invalid	Lack of desired induction in negative emotional class
8	Male	20	19	7.8	7.4	2.8	3	Valid	–
9	Male	26	9	7.4	7	3.4	5.4	Invalid	Lack of desired induction in negative emotional class
10	Female	23	9	6.8	6.6	3.8	3.2	Invalid	Lack of desired induction in negative emotional class
11	Female	25	22	7.8	8	4.5	3	Invalid	Beck depression (22 > 21)
12	Female	27	1	8.6	8.6	2	1.2	Valid	–
13	Female	29	9	6	6	2	1.2	Valid	–
14	Male	26	8	8	8	1.8	1.6	Valid	–
15	Female	25	12	–	–	–	–	Invalid	Motion noise
16	Male	27	0	7.4	8	1.8	2	Valid	–

**Table 2:** Sequence and type of music used for positive (P), and negative (N) emotional stimulation^43^.

Used song	Type of induced emotion	Symbol
Prelude to Isfahan By Mohammad Reza Lotfi	Negative	N1
Six and eight Azeri	Positive	P1
Homayoun Preface By Faramarz Payvar	Negative	N2
Six and eight Azeri	Positive	P2
Six and eight ports	Positive	P3
Afshari piece By Sohrab Pournazeri	Negative	N3
Prelude to Isfahan By Mohammad Reza Lotfi	Negative	N4
Six and eight Persian	Positive	P4
Precursor Dashti By Hossein Alizadeh And Kayhan Kalhor	Negative	N5
Six and eight ports	Positive	P5

## Simulation results

The Brainstorm toolbox in MATLAB R2019a was used to calculate the active brain regions by sLORETA method. The results of this method are used as the initial value for the Bayesian model based on Bernoulli-Laplace prior. A server with an NVIDIA 1080TI GPU and an Intel Core i7 CPU is intended to implement the proposed algorithm in Tensorflow 2.0.0 in Python programming language. The results of the proposed algorithm for automatic detection of emotions are presented in the continuation of this section. In this study, unlike many studies, the evaluation results of the proposed method for inducing emotion with both music and image are presented, so to fairly compare the proposed and the existing state-of-the-art methods; we implement both categories of approaches on our recorded data, SEED and DEAP datasets. The sources with less than 50% of a subject's maximum power are eliminated to reduce the computational cost of algorithm.

The proposed method is evaluated in two subject-dependent and subject-independent scenarios. In the subject-dependent scenario, 4 out of 10 trails are randomly considered as a training set and the remaining 6 experiments are considered as a testing set. In addition, in a subject-independent scenario, the data of 40% subjects are used for training and 40% subjects for testing and 20% subjects for validation of proposed method. Finally, the average accuracy performance of the proposed method is reported to all subjects.

**Figure 9:**
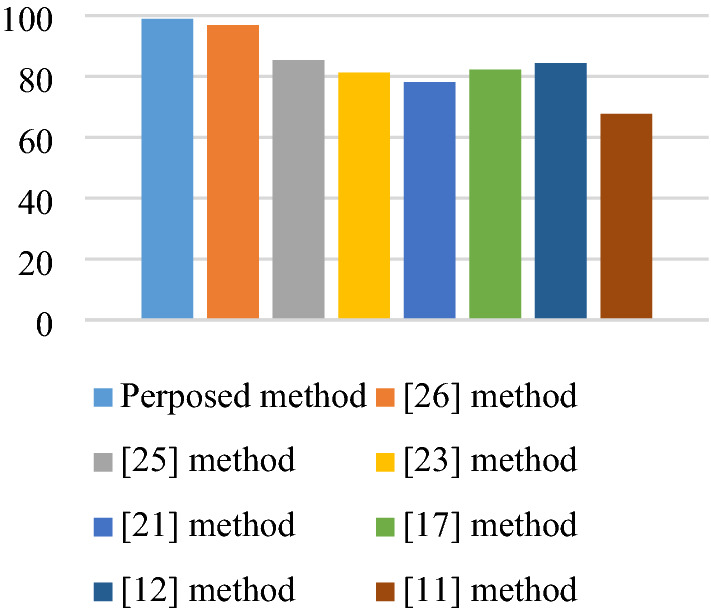
Subject-dependent scenario accuracy of the proposed method as well as the methods presented in methods^11,12,17,21,23,25,26^ for recorderd EEG.

**Figure 10:**
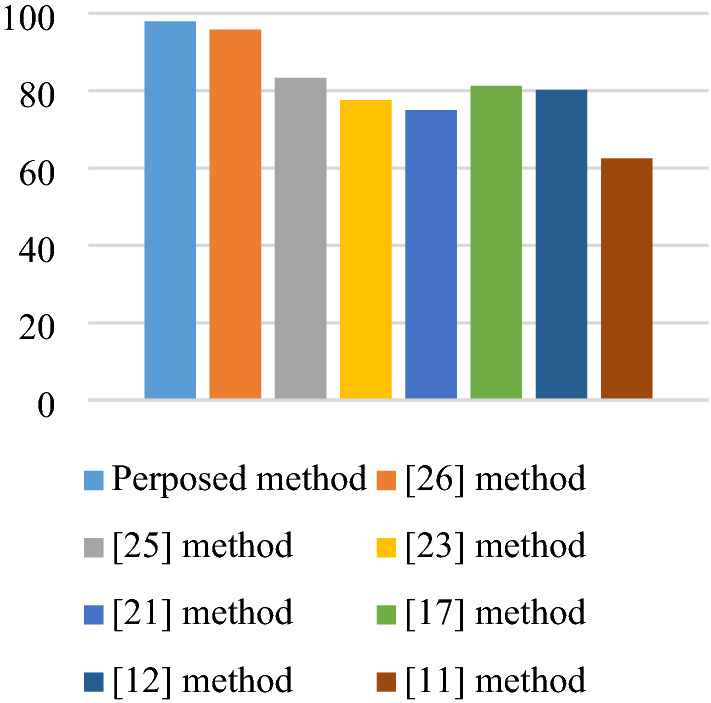
Subject-independent scenario accuracy of the proposed method as well as the methods presented in^11,12,17,21,23,25,26^ for recorderd EEG.

The accuracy of the subject-dependent scenario of the proposed method and the existing methods^11,12,17,21,23,25,26^ are compared in Fig. 9. The lowest accuracy in this comparison is related to the method in^11^ with 67.7%. However, for the method^26^, the average accuracy is 96.87%. It can be seen that in all subjects, the highest accuracy is related to the proposed method with 98.95%. The proposed method and the methods^11,12,17,21,23,25,26^ in Fig. 10 were compared in the form of subject-independent scenario. As can be seen, the best accuracy of the subject-independent scenario is related to the method in^26^ with 95.83%. However, our proposed algorithm in this scenario gives 97.91% accuracy.

These results indicate the robustness of the proposed algorithm against cross-subject variations. According to the results, the accuracy of subject-independent scenario is less than the accuracy of subject-dependent scenario, the reason for this issue is the use of unseen data to test the algorithm in a subject-independent scenario. It is clear from the results that the accuracy of the proposed algorithm in both subject-dependent and subject-independent scenarios is better than the methods available in^11,12,17,21,23,25,26^. The evaluation results of the proposed method and the existing methods are presented for the SEED and DEAP dataset in Tables 3 and 4, respectively. The accuracy of the subject-independent scenario is 98.51% and 98.32% and the subject-dependent scenario is 99.25% and 98.96% for our proposed method on the SEED and DEAP dataset, respectively. The highest accuracy for subject-dependent scenario and the subject-independent scenario for the proposed method^26^ among the available methods have been calculated as 98.51% and 97.77%, respectively. The accuracy obtained for our proposed method is greater than other methods. As shown in Table 5, when Bernoulli-Laplace-based Bayesian model is used for source localization, the accuracy of the proposed algorithm is higher than when sLORETA is used. According to Table 5, if CNN classifier is used instead of DGCNN, the accuracy of the proposed algorithm be lower.

**Table 3:** Classification accuracy and details of the proposed method and deep neural network approaches which used raw EEG signals^11,12,17,21,23,25,26^ for the SEED dataset.

Subject-dependent/independent	Methods	Used signals	Accuracy (%)
Subject-dependent	Jin et al.^26^ (2020)	Raw EEG	**98.51**
	Zhong et al.^25^ (2020)	Raw EEG	92.58
	Khare et al.^23^ (2020)	Raw EEG	91.10
	Wang et al.^21^ (2020)	Raw EEG	89.62
	Song et al.^17^ (2018)	Raw EEG	84.43
	Chen et al.^12^ (2020)	Raw EEG	81.47
	Padilla et al.^11^ (2016)	Raw EEG	62.95
	Proposed method	EEG source signal	**99.25**
Subject-independent	Jin et al.^26^ (2020)	Raw EEG	**97.77**
	Zhong et al.^25^ (2020)	Raw EEG	88.88
	Khare et al.^23^ (2020)	Raw EEG	81.48
	Wang et al.^21^ (2020)	Raw EEG	74.07
	Song et al.^17^ (2018)	Raw EEG	72.59
	Chen et al.^12^ (2020)		70.37
	Padilla et al.^11^ (2016)		59.25
	Proposed method	EEG source signal	**98.51**

Significant values are in bold.

**Table 4:** Classification accuracy and details of the proposed method and deep neural network approaches which used raw EEG signals^11,12,17,21,23,25,26^ for the DEAP dataset.

Subject-dependent/independent	Methods	Used signals	Accuracy (%)
Subject-dependent	Jin et al.^26^ (2020)	Raw EEG	97.91
	Zhong et al.^25^ (2020)	Raw EEG	92.05
	Khare et al.^23^ (2020)	Raw EEG	90.88
	Wang et al.^21^ (2020)	Raw EEG	89.19
	Song et al.^17^ (2018)	Raw EEG	83.20
	Chen et al.^12^ (2020)	Raw EEG	79.81
	Padilla et al.^11^ (2016)	Raw EEG	58.36
	Proposed method	EEG source signal	**98.96**
Subject-independent	Jin et al.^26^ (2020)	Raw EEG	97.52
	Zhong et al.^25^ (2020)	Raw EEG	87.24
	Khare et al.^23^ (2020)	Raw EEG	80.72
	Wang et al.^21^ (2020)	Raw EEG	72.39
	Song et al.^17^ (2018)	Raw EEG	71.48
	Chen et al.^12^ (2020)		69.66
	Padilla et al.^11^ (2016)		57.94
	Proposed method	EEG source signal	**98.31**

Significant values are in bold.

**Table 5:** Subject-independent classification accuracy and details of the proposed method and hybrid method and CNN classifier.

EEG source localization method	The type of classifier	SEED dataset (%)	Recorded dataset (%)	DEAP dataset (%)
sLORETA	CNN	95.56	93.75	95.18
sLORETA	DGCNN	97.78	97.50	97.65
Bernoulli–Laplace-based Bayesian model	CNN	96.67	95.31	96.48
Bernoulli–Laplace-based Bayesian model	DGCNN	98.51	97.91	98.31

## Discussion and conclusion

In this study, we propose an algorithm based on DGCNN and EEG sources to recognize emotions. A mapping of scalp sensors to brain sources is performed to extract the pattern of each emotion using Bayesian model based on Bernoulli-Laplace prior. The results of sLORETA method is used for initialization of this model. In the proposed method, a DGCNN is used to classify emotion-based EEG in which the sources of the Bayesian model based on Bayesian model based on Bernoulli–Laplace prior method are considered as underlined graph signals. Finally, emotional EEG signals are divided into negative and positive emotional classes using this approach. The proposed method is compared with existing standard methods in subject-independent and subject-dependent experiments on our emotional EEG dataset, DEAP and the SEED dataset.

Feature extraction from EEG data in all previous methods is a major challenge. In this study, to solve this problem, the spatio-temporal information of emotional EEG sources is encoded in a graph. The DGCNN algorithm is then used to classify these graphs. Using purposed approach, acceptable accuracy for the data is obtained without the need to design the feature extraction process. According to the results, the proposed technique has made the brain areas involved in emotions processing more focused. Significant differences can be seen in the areas involved during the induction of positive and negative emotions. This issue significantly increases the accuracy of the emotion classification. Another point in the proposed method is the updating of the adjacency matrix in DGCNN algorithm, which in itself improves the emotion classification accuracy.

Increasing the number of electrodes used to record the signal based on the results of previous studies in the field of EEG signal processing^46^, improves classification accuracy. However, the problem is that it is costly and time-consuming to use high-density EEG sensor arrays in a clinical or field environment. In this study, we use the source localization technique to increase spatial information in EEG recordings. The spatial resolution of EEG recordings can be expanded by increasing the number of sources. These sources contain good spatio-temporal information. According to the concepts mentioned in the results section, the accuracy of the subject-dependent scenario and the subject-independent scenario for our proposed method are 99.25% and 98.51%, respectively. These accuracies are greater than the values obtained in existing state-of-the-art methods.

The use of video or music video to induce emotions, in addition to the areas related to emotion processing, also involves the visual and memory areas^11,12,17,21,23,25,26^. Considering the results of emotion induction using music in this study and this issue, it is clear that auditory induction can be an easier and more appropriate way to induce emotions. In this study, the weight of each graph edge is determined by calculating the correlation between the graph signal sources. In future studies, another feature of graph signals can be used as a criterion to calculate the weight of edges.
